# Is the Supercourse useful for Latin America?

**DOI:** 10.5195/cajgh.2012.9

**Published:** 2013-02-21

**Authors:** Nicolas Padilla-Raygoza, Faina Linkov, Eugene Shubnikov, Ron E. LaPorte, Rosalina Diaz-Guerrero

**Affiliations:** 1 Departament of Nursing and Obstetrics, Division of Health Sciences and Engineering, Campus Celaya Salvatierra, University of Guanajuato; 2 Division of Cancer Prevention and Population Science, University of Pittsburgh Cancer Institute; 3 Institute of Internal Medicine, Novosibirsk, Russian Federation; 4 Department of Epidemiology, Graduate School of Public Health, University of Pittsburgh Cancer Institute

**Keywords:** Supercourse, education, Latin America

## Abstract

**BACKGROUND:**

The success of the Supercourse showed that the effort was needed in Latin America. But would a Spanish language version be better for the region?

**METHODS:**

Google Analytics was used to determine website usage. A custom evaluation form was created to get user feedback on the usefulness of both the English language and Spanish language Supercouse lectures.

**RESULTS:**

Over a year’s span from June 2009 to June 2010 there were 257,403 unique visits and 448,939 page views. The overall average rating of lectures was 4.87 with the Spanish language lectures getting even higher ratings.

**CONCLUSION:**

Supercourse lectures in Spanish were a great success in Latin America. This success shows the need for this information and similar success could be found in Central Asia.

## Introduction

The Global Health Network Supercourse project was launched in the late 1990’s to help with the difficult task of providing high quality teaching materials to instructors of the world who wanted to teach about global health and prevention (www.pitt.edu/~super1). The Supercourse group argued that cooperation in higher health education was needed to prevent duplicate efforts and a waste of resources.^1^ In addition, it has been found that the vast majority of the materials used in classes were at least 5 years old, which is far behind the fast pace of science. Thus, the Supercourse was developed in order to speed up the translation of research information from labs to classrooms. This initiative was originally supported by NASA and the National Institutes of Health and it had great success, reaching 174 countries of the world.

In the mid 2000’s the Supercourse effort started to spread to the countries of Central Asia as well as Mexico. As these efforts spread in these two very different geographic areas, we found that the challenges were surprisingly similar: a lack of English skills among local faculty members, a lack of time, and simply low awareness about this global effort. Thus, we view the development of the Supercourse in Mexico and Central Asia as sister efforts.

The Supercourse model empowers public health teachers worldwide by offering more than 4700 high quality lectures free of charge, distributing them through the Internet and via DVDs, with the invitation to join and expand the network. One of the main goals of the Supercourse is to help professors and instructors diminish the time spent developing a lecture for his or her class through the availability of the slides of lectures in the Supercourse.^2^ Thus, a teacher could use 5 slides from an expert in cancer, 10 from an expert in diabetes, and 8 of their own. The “recycling” of slides markedly improves the quality of lectures, as the slides come from the world’s experts, as well as the efficiency, as one does not have to make slides and lectures from scratch. Moreover, as the majority of the slides are developed in leading academic institutions, they are quite up to date.

The Supercourse remains a very active and highly visited website today. In the first step of project development, the Supercourse team established a network of collaborators, then began to collect lectures from recognized authors from the University of Pittsburgh. Little by little, it grew until it reached approximately 50,000 faculty members in 174 countries, with Nobel Prize laureates in Medicine donating several lectures to the Supercourse.^3^ Through collaboration with the network of scientists in Mexico, the Supercourse website and its materials became accessible to Spanish speaking faculty members. Under the direction of Nicolas Padilla, a new version of the Supercourse has successfully been built: the Latin American Supercourse (http://www.feoc.ugto.mx/super/curso.php).

The initial success of the Supercourse was demonstrated by the number of daily site hits and the search tools in Google®, where the lectures from the Supercourse are in the first ten among millions of results, and at one point receiving recognition as one of the top webpages in the area of health by PCMagazine as well as Science Magazine.^3^

Eight years ago, the School of Nursing and Obstetrics of Celaya, from the University of Guanajuato, Mexico, began to translate some lectures from English to Spanish in order to overcome the language barrier and to ensure that global health knowledge reaches more health professionals in Spanish speaking countries, including those of Latin America.

Health professionals in Latin American countries have little or no access to continuing education materials in the field of public health because of high costs, the need to travel, a lack of English language skills, etc. This is especially true when it comes to obtaining training in global health and prevention, as medical schools traditionally focus on clinical work. The Supercourse and the Latin American Supercourse overcome this difficulty because materials are updated and offered via the Internet free of charge. The Supercourse uses low bandwidth technology, allowing for easy access in regions with slow or expensive Internet connections.

Because of the growing interest in Spanish language content, the next logical step was to launch the Latin American Supercourse on Epidemiology, Internet and Global Health on May 3th, 2007.^4^ Lectures chosen for translation into Spanish were those that were identified to be the most valuable for Spanish speaking faculty members in Celaya, Mexico. The goal of this paper is to report the progress of the development of the Latin American Supercourse and to explore preliminary data about its usage. It is our belief that our findings will have very interesting implications for the countries of Central Asia and that our study can encourage the development of similar assessments in the region.

## Methods

### Website utilization: Google Analytics

Google Analytics (GA) has been used to collect basic information about the utilization of the Supercourse website in terms of number of visits per country, per language, etc. GA is a free service offered by Google that generates detailed statistics about the visitors to a website. GA is currently in use at around 57% of the 10,000 most popular websites. GA can track visitors from all referrers, including search engines, display advertising, pay-per-click networks, email marketing, and digital collateral such as links within PDF documents.

### Lecture evaluation: Lecture Review Form

**Figure 1:**
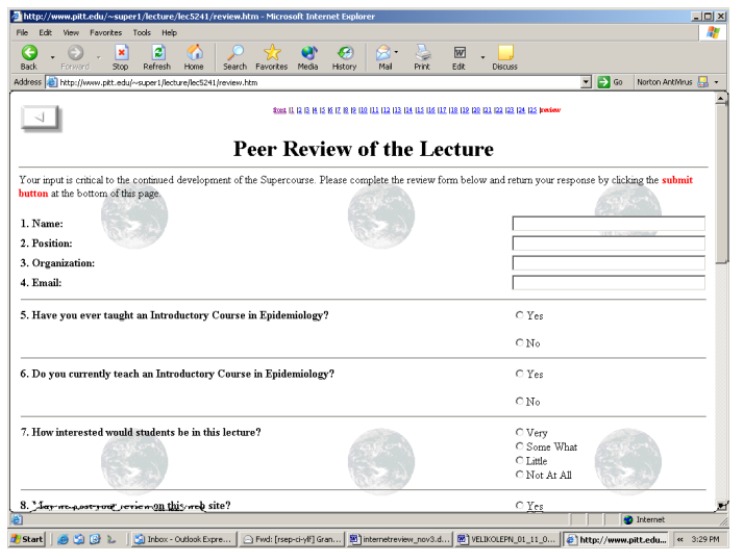
Lecture Review form, Supercourse Project

The lecture review forms utilized in this effort (see Figure 1) have the following questions: name, position, organization, e-mail address, have you ever taught an introductory epidemiology course (yes or no), rate the lecture on content, presentation, relevance, and overall rating (the rating scale for these last four items: 5 = excellent, 4 = above average, 3 = average, 2 = below average, 1 = poor). In May 2004, Dr. Songer suggested that an “expectation” rating of the lecture may provide many important insights into quality measurement for the Supercourse. In August 2004, an additional question was added to all of the Supercourse peer review forms: “How does the quality of the lecture compare with your expectations about it?” (The rating scale for this item also utilizes Likert scales: 5 = Well above what I expected, 4 = Above what I expected, 3 = Same as expected, 2 = Somewhat below what I expected, 1 = Well below what I expected.)

## Results

Using Google analytics® from June 10th, 2009 to June 10th, 2010, there were 257,403 unique visits to the main webpage of the Supercourse, with 448,939 page views. This means that on average, a visitor accessed 1.74 lectures. Table I demonstrates the number of visits to the Supercourse webpage from 212 countries/territories; the visits were mainly from United States of America, India, and United Kingdom. It is interesting to point out that the Biostatistics Course by Nicolas Padilla, Lecture #15 on Correlation, is the 5th most visited lecture in the Supercourse as of October 2010.

Table II presents the number of accesses of the Supercourse webpage by countries and languages. It is important to point out that most popular languages were English and Spanish. The number of visits is a very low estimate of the usage of the Supercourse, as these numbers include visits only to the main server in Pittsburgh. Due to technical limitations, it is not possible to assess the number of visits to the mirror server sites, the number of lectures downloaded in PowerPoint format, and the number of lectures presented in front of classrooms.

Lecture evaluations filled out in Spanish can be used as a surrogate measure of interest of Spanish language speakers in certain areas. Based on this assessment, it appears that the ten most interesting topics to Latin American faculty members include: addictions, diabetes, global warming, disasters and Just-in-Time lectures, statistics, nursing, infectious diseases, epidemiology, public health, and cancer. This appears to be somewhat similar to the interest of English language faculty members, who are also interested in Just-in-Time lectures, cancer, and statistics, while the difference between these groups is that English speaking faculty members appeared to be less interested in infectious disease. For the 3,233 evaluations we obtained, content had a mean score of 4.82±0.61, relevance had a mean score of 4.84±0.55, and presentation had a mean score of 4.78±0.69, with the overall average rating being 4.87±0.47 and the expected quality average rating being 4.80±0.58. Clearly the lectures are most appreciated. The mean scores for lectures translated into Spanish were at least 0.5 points higher than scores for the main Supercourse lectures in English.

The Latin American Supercourse was divided into areas (see Table III) to make searching easier. In three years, the Supercourse had almost 30,000 webpage accesses to the front page alone, and the Latin American Supercourse database had faculty members from almost all Latin American countries. Each month, members of the network received a newsletter from the Latin American Supercourse with news about new lectures, changes in the lecture of the week, or updates about new important lectures, such as those for Influenza A (H1N1) or hurricanes.^5^,^6^ These messages were regularly sent to over one thousand of e-mails contacts from the Supercourse Latin American Network.

## Conclusions

Large numbers of public health instructors, especially Spanish speaking instructors, are using the Supercourse lectures as a source of information from the world’s leaders in public health and medicine. The efforts of the Supercourse and the Latin American Supercourse teams are giving “fruit” in the form of better, up to date education for public health professionals. The selection of translated lectures in the Latin American Supercourse potentially represents gaps in the existing materials available to Mexican faculty members, such as biostatistics, infectious disease epidemiology, and just-in-time knowledge on disasters.

The challenges involved in the development of the Latin American Supercourse include identifying collaborators and identifying a supporting mechanism for sustainable development of this effort. Another big challenge is that many faculty members of the Latin American based institutions do not have the websites of their schools available on the Internet, and thus they are difficult to find for initial contact. Additionally, many faculty in Latin American countries do not speak English, making international collaboration hard to initiate. As we mentioned in our introduction, similar challenges have been found in Central Asia.

The parent Supercourse and the Supercourse translations into Spanish are having a great impact on health professionals from Latin American countries, demonstrated by the number of webpage accesses and page views. Interest in the Spanish language Supercourse is also demonstrated by evaluations, which are scored even higher than English language Supercourse lectures. It is likely that the Latin American Supercourse lectures are teaching more students about Global Health and Prevention than any other lectures, especially in Latin American countries. The Latin American Supercourse will also continue to work on developing advanced quality control methodologies, building on the existing publications in this area.^7^–^11^ Expansion of the Latin American Supercourse is an important and much needed effort.

Since the situations in Latin America and Central Asia are similar, we would argue that it would be important to develop more efforts into getting the Supercourse translated into Russian and the other languages utilized in Central Asia. The popularity and high ratings of the translated lectures show that this information is desired but unavailable to those who do not speak English. These translation efforts would greatly improve the reach of public health information in the region and globally.

## Figures and Tables

**Table I:** Visits to Supercourse Webpage by Country/Territory

Country	n	%
United States of America	110,299	42.8
India	14,875	5.8
United Kingdom	13,595	5.3
Canada	10,845	4.2
Australia	6,470	2.5
Philippines	4,781	1.9
Malaysia	3,937	1.5
Pakistan	3,568	1.4
Mexico	3,305	1.3
Egypt	3,224	1.3
Other	82,504	32.0
Total	257,403	100.00

Source: Google Analytics, from June 10th, 2009 to June 10th, 2010

**Table II:** Languages of Lectures from the Supercourse Webpage

Language	Country	n	%
English	United States of America	206,348	80.2
Spanish	Latin American countries	7,133	2.8
English	Great Britain	6,602	2.6
English	Other countries	4,267	1.7
Chinese	Taiwan	2,934	1.1
Chinese	China	2,782	1.1
French	France	2,610	1.0
German	Germany	2,412	0.9
Spanish	Spain	2,344	0.9
Russian	Russia	2,207	0.9
Other	Other	17,764	6.8
Total		257,403	100.0

Source: Google Analytics, from June 10th, 2009 to June 10th, 2010

**Table III:** Areas of concentration for Latin American Supercourse and numbers of lectures available in these areas

Area	Number of lectures by year						Total	
	2007–2008		2009		2010			
	O	T	O	T	O	T	O	T
Addictions	6	9	2	0	1	0	9	9
Cancer	0	11	0	1	0	0	0	12
Global Warming	1	2	1	0	0	0	2	2
Disasters	0	15	0	2	0	0	0	17
Diabetes	2	3	0	0	0	0	2	3
Cardiovascular diseases	10	3	0	1	0	0	10	4
Infectious diseases	5	10	1	0	0	0	6	10
Nursing	104	0	4	2	0	0	108	2
Statistics	0	8	16	0	0	1	16	9
Epidemiology	1	81	2	1	0	1	3	83
Nutrition and obesity	0	8	0	4	0	0	0	12
Global health	3	11	2	3	0	0	5	14
Maternal and childhood health	5	62	0	0	5	0	10	62
Public health	9	15	2	0	0	0	11	15
Supercourse, internet, and technology	11	27	0	1	0	2	11	30
Just in Time	0	25	0	1	0	3	0	29
Total	157	290	30	16	6	7	193	313

Source: LatinAmerican Supercourse webpage (http://www.feoc.ugto.mx/super/curso.php)

O = Original

T = Translation
